# Reducing health inequities facing boys and young men of colour in the United States

**DOI:** 10.1093/heapro/daaa148

**Published:** 2020-12-27

**Authors:** James A Smith, Daphne C Watkins, Derek M Griffith

**Affiliations:** 1 Freemasons Centre for Male Health & Wellbeing-Northern Territory, Menzies School of Health Research, Charles Darwin University; 2 Curtis Center for Health Equity Research & Training, School of Social Work, University of Michigan; 3 Center for Research on Men's Health, Vanderbilt University

**Keywords:** inequities, men’s health, health promotion, race, ethnicity

## Abstract

Health promotion research and practice consistently reveals that people of colour in the USA face multiple structural and systemic health and social inequities as a direct consequence of racism and discrimination. Recent scholarship on equity and men’s health has highlighted the importance of gender—specifically concepts relating to masculinities and manhood—to better understand the inequities experienced by men of colour. A sharper focus on the intersection between race, gender and life stage has also emphasized the importance of early intervention when addressing inequities experienced by boys and young men of colour (BYMOC). This has led to an expansion of health promotion interventions targeting BYMOC across the USA over the past decade. Many of these health promotion strategies have attempted to reduce inequities through action on the social determinants of health, particularly those that intersect with education and justice systems. Reflecting on these developments, this commentary aims to discuss the challenges and opportunities faced by the health promotion community when attempting to reduce health and social inequities experienced by BYMOC. In doing so, the solutions we identify include: strengthening the evidence base about effective health promotion interventions; reducing system fragmentation; promoting connectivity through networks, alliances and partnerships; reducing tensions between collaboration and competition; changing the narrative associated with BYMOC; acknowledging both inclusiveness and diversity; addressing racism and intergenerational trauma; and committing to a national boys and men’s health policy. We encourage health promotion researchers, practitioners and policy-makers to adopt these solutions for the benefit of BYMOC in the USA.

## INTRODUCTION

This article aims to explore the challenges and opportunities associated with reducing health and social inequities experienced by boys and young men of colour (BYMOC) in the USA, specifically African American, Native American and Latinx boys and young men. It is based on a recent Fulbright project undertaken by J.S. in collaboration with D.W. and D.G. from February to June 2020. The project involved scanning current academic and grey literature on this topic using a range of academic databases including PubMed, Scopus, PsychINFO, Informit, Web of Science and Google Scholar from the last 15 years (2006–20). Key search terms included health promotion, health equity, youth, men, race, ethnicity and colour/color, or variants thereof. Feedback from a series of informal discussions with more than 40 practitioners, researchers and policy-makers from across the USA engaged in work with BYMOC was also used to identify pertinent grey literature including evaluation reports and literature reviews and evidence scans. These informants were drawn from local, state and national organizations supporting the health and wellbeing of BYMOC, and primarily included practitioners and policy-makers from public health, education, justice and employment backgrounds; and scholars from research centres dedicated to men’s health, health equity, and race and health. An ethics exemption was obtained from the Vanderbilt University Institutional Review Board (IRB) (#200280) with reciprocal ethics exemptions obtained from the University of Michigan IRB (HUM00178288), and the Northern Territory Department of Health and Menzies School of Health Research Human Research Ethics Committee (HREC 2020-3648) This article is not intended to be empirical and it does not include the explicit findings from analyzing the interviews. Rather it is reflective commentary—based on the current evidence base—which aims to promote scholarly debate and discussion.

### Addressing health and social inequities among people of colour

Black, Indigenous, and People of Colour (BIPOC) in the USA have faced multiple health and social inequities for generations, particularly those relating to race and ethnicity (Jones *et al.*, 2012; Gilbert *et al.*, 2016; Buyum *et al.*, 2020). For the purposes of this article, we define health and social inequities as those that are preventable and considered to be unfair and unjust. These structural inequities have spanned, but are not limited to, health, education, justice, housing, transportation and employment sectors (Bailey *et al.*, 2017). While health promotion scholarship emphasizes the urgency of action to address the impacts of racism, trauma, and other social and cultural determinants of health, ameliorating health inequities by focusing on social determinants of health have not been the primary focus of efforts to date. In particular, comprehensive strategies to address structural and systemic racism have been sparse. Notably recent events relating to police brutality and murders of BIPOC, particularly young Black males, has heightened awareness of racism in the USA (Gilbert and Ray, 2016; Hartfield *et al.*, 2018). Such events have provoked strong civil society action and calls for change at national and global levels in the form of protesting and demonstrations; and helped to advance the mission of the Black Lives Matter movement (García & Sharif, 2015). Yet, it is evident that much more work needs to be done, and that this needs to be driven by a much stronger social determinants of health lens.

### Addressing health and social inequities faced by boys and young men of colour

The health and social inequities experienced by BYMOC are diverse and are noted across a range of sectors. It is beyond the scope of this article to explain these in detail. However, at a macro-level, there is robust evidence to suggest that the following inequities exist among this population:

- Challenges associated with health and social service access, which impinge on help-*seeking* practices and health service use (Vogel *et al.*, 2011; Planey *et al.*, 2019).
- Poor education outcomes, including low levels of participation, achievement and completion across all levels of the education system (White, 2009; Addis and Withington, 2016; Ferguson, 2016; Voisin and Elsaesser 2016; Cook *et al.*, 2017); high levels of disengagement and suspension (Fenning and Rose, 2007; Losen 2011; Ferguson, 2016; Godsil, 2017); and low levels of postsecondary education and career aspiration.
- Over-representation in the child welfare system, with clear evidence this impacts lifelong education, employment and incarceration trajectories (Greenfield, 2010; Cook et al., 2017)
- High rates of incarceration (Williams and Bergeson, 2019)
- Poor job attainment and retention, and high rates of unemployment (Bird, 2016)
- Challenges associated with accessing and retaining safe and secure housing; and homelessness (Gattis and Larson, 2016)
- High rates of risky health practices—including those relating to smoking (Freedman et al., 2012); unsafe sex (Crosby et al., 2016; Aduloju-Ajijola and Payne-Foster, 2017); alcohol and substance misuse (Chartier et al., 2011); and violence (Chartier et al., 2011; Rich, 2016).
- Poor mental health (Prevention Institute, 2014) and high rates of suicide ideation and suicide (Lindsey and Xiao, 2019).

We acknowledge this only provides a partial snapshot of some of the inequities BYMOC face.

Importantly, public health and health promotion research on health and social inequities has increasingly paid attention to intersections between race, gender and life stage (Gilbert *et al.*, 2016; Griffith, 2020). In particular, scholarship on equity and men’s health has frequently highlighted the importance of understanding gender—that is, concepts of masculinities and manhood—within the context of disadvantage experienced by men of colour (Jones *et al.*, 2012; Metzl, 2013; Watkins and Griffith 2013; Griffith 2015, 2018; Thorpe *et al.*, 2015; Gilbert *et al.*, 2016; Smith *et al.*, 2020). For purposes of clarity:

…manhood is a relational construct that highlights how age shapes the meaning of masculinity, and the way men prioritize performing or demonstrating that they are indeed men (e.g., not boys, not feminine). Manhood also implicitly offers a set of characteristics and virtues that adult males use to demonstrate and embody key gendered, racialized and class-bound values and goals [(Griffith, 2015), p. 288].

In addition, the adoption of a life-course approach has emphasized the importance of investing in health promotion and prevention efforts in the early years of life to reduce the cumulative and intergenerational impacts of health and social inequities faced by BYMOC (Jones *et al.*, 2012; Griffith 2015; Thorpe *et al.*, 2015; Cunningham and White, 2019). That is, health promotion strategies tailored to the unique needs of BYMOC, and which account for environmental, geographical and contextual differences between different groups of BYMOC, are needed now more than ever (Rawlings, 2015; Cunningham and White, 2019; Rigg *et al.*, 2019).

## DISCUSSION

### Understanding the challenges and opportunities for reducing health and social inequities among BYMOC

In the following section, we provide brief commentary about the challenges and opportunities that face the health promotion community in the USA to better promote health and social equity among BYMOC. The intention is to provide a strengths-based and solution-focused narrative that can inform affirmative health promotion research, practice and policy responses.

### Strengthening the evidence base about effective health promotion interventions

Over the past decade, there have been numerous literature reviews, field scans and evidence syntheses that have described the challenges that confront BYMOC. Much of this lies within the grey literature as commissioned reports produced for national organizations and large philanthropic foundations. This has spanned topics relating to family and community health (Randolph-Back, 2006; Astone *et al.*, 2015; Philpart *et al.*, 2015); education (Addis and Withington, 2016; Ferguson, 2016; Voisin and Elsaesser 2016; Godsil, 2017); employment and training (Spaulding *et al.*, 2015; Bird, 2016); criminal justice (Liberman and Fontaine, 2015; Cook *et al.*, 2017); healing and trauma (Rich, 2016); mental health (Cook *et al.*, 2017; Prevention Institute, 2014); economic opportunity (Spaulding *et al.*, 2015; Spievack *et al.*, 2020); and achievement, life chances and success (White, 2009; Wimer and Bloom, 2014; Robert Wood Johnson Foundation, 2015). While these reports are useful for outlining the challenges BYMOC face, relatively few present evidence-based strategies that can be adopted to change the underlying health and social issues identified. In short, a significant ‘knowledge-behaviour’ gap exists. Even when turning to research and evaluation papers focused on specific interventions targeting BYMOC, these are generally limited to specific topics such as sexual health (Baker *et al.*, 2012; Crosby *et al.*, 2016) and mental health (Watkins *et al.*, 2017, 2020; Goodwill *et al.*, 2018), but inadequate attention has been paid to other health issues like chronic diseases, oral health and well-being. This suggests that research and interventions need to expand their focus to look more holistically at the health of BYMOC. This includes adopting a broader social determinants of health perspective. The health promotion community is well positioned to support this undertaking. Stronger research–practice partnerships focused on improving the health and well-being of BYMOC are clearly needed.

### Reducing system fragmentation

There are numerous national organizations and programmes focused on improving the lives of BYMOC including: My Brother’s Keeper; Campaign for Black Male Achievement; Forward Promise; National Compadres Network; Making Connections; National Black Men’s Health Network; Coalition of Schools Educating Boys of Colour; Executives’ Alliance for Boys and Men of Colour; and Research Integration Strategies Evaluation (RISE) for Boys and Men of Colour. These examples are indicative only—as there are many other not-for-profit organizations and foundations that are also achieving great outcomes. However, in our view there is insufficient investment, and a lack of health and social policy direction, to adequately address the magnitude of the public health crisis facing BYMOC in the USA.

The individual contributions they each make are important, but without coordination there is potential for unnecessary duplication. Indeed, many of the stakeholders contacted as part of the Fulbright project revealed that system fragmentation was a major issue. That is, there was a broad recognition that organizations and programmes targeting BYMOC set their own agendas and missions without necessarily considering, or dovetailing with, other similar organizations or the broader men’s health landscape in the USA. If resourced appropriately, there is room for organizations serving BYMOC to connect more purposefully with other mainstream men’s health organizations, such as the Men’s Health Caucus of the American Public Health Association, the Men’s Health Network and the Partnership for Male Youth Health. This does not mean leadership and campaigning associated with BYMOC is lacking, or that mainstream men’s health organizations are better positioned to advocate on behalf of BYMOC. Rather it suggests that all organizations interested in reducing health inequities among BYMOC could co-develop a better co-ordinated and more integrated national plan of action for this population. Government support would be advantageous, and a national policy response would be a sensible course of action. This could potentially be mirrored on national men’s health policy responses adopted in Australia and Ireland, where there have been foci on equity and marginalized populations; increased investment in community-based health promotion and prevention efforts; and a commitment to building a stronger evidence base to inform men’s health practice (Richardson and Smith, 2011; Richardson *et al.*, 2019). In any case, reducing system fragmentation would help to pool finite resources, enhance partnership development and promote information sharing across the USA in ways that will benefit BYMOC.

### Promoting connectivity through networks, alliances and partnerships

There are multiple networks, alliances and partnerships that support BYMOC. These span youth-, men’s health-, Native American-, and African American-focused organizations. However, the connectivity between them is primarily reliant on personal and professional relationships between enthusiastic and committed individuals. While there is some evidence of collaboration, particularly in relation to professional development opportunities such as workshops and webinars, and through joint sponsorship of local level community activities, there is minimal evidence of genuine long-term partnerships at programmatic and policy levels. There is also a lack of overarching governance structures to facilitate relationship and leadership development at a more systemic level. That is, further work is required to mobilize and coordinate across the critical mass of stakeholders that are working with BYMOC. Akin to the discussion about system fragmentation above, mechanisms to pool resources and scale efforts to serve BYMOC in more integrated ways is needed, particularly when addressing social determinants of health. This would provide a more purposeful strategy to address structural and systemic inequities experienced by BYMOC.

### Reducing tensions between collaboration and competition

In many health promotion contexts, there is a tension between collaboration and competition. This exists in both research and practice domains. There are expectations to collaborate with colleagues and communities, particularly in relation to addressing equity through action and examining social determinants of health. In research settings, inter- and trans-disciplinary studies are celebrated; and research–policy–practice partnerships considered the hallmark of effective knowledge translation efforts. In practice settings, intersectoral action is welcomed; whole-of-government processes encouraged; and Health-in-All-Policies approaches advocated. Yet, to attract funding, and for organizations to remain viable, they must compete with one another for limited resources. This rhetoric has been particularly evident in work relating BYMOC. A number of not-for-profit organizations have successfully developed business models that help to sustain programme operations within local or state-based contexts, such as the Adonai Center for Black Males in Pittsburgh; Beats Rhymes and Life Inc. and the Ever Forward Club both in Oakland; the Tennessee Men’s Health Network; and the Young Men’s Clinic in Boston. Yet, others have not. In particular, some of the larger national organizations have struggled to sustain their existence within a changing socio-political context and a hyper competitive funding environment. The Campaign for Black Male Achievement is currently sun setting; My Brother’s Keeper has transitioned from the government to the Obama Foundation in a substantially scaled back form; and RISE for Boys and Men of Colour has ceased. Arguably, these initiatives have all been an important part of the national fabric to reduce health and social inequities among BYMOC, whether through synthesizing and sharing information about the current evidence base; building leadership capacity; or providing a framework for action on the social determinants of health. However, this ebb and flow of national leadership impacts the ability to address structural and systemic barriers facing BYMOC over the long term. Indeed, the rise and fall of prominent BYMOC initiatives and organizations lead to piecemeal health promotion responses that perpetuate, rather the ameliorate, inequities among this marginalized population. The intent is not to apportion blame. Rather to emphasize that more strategic and large-scale investments are required to purposefully respond to the immediate needs of BYMOC.

### Changing the narrative associated with BYMOC

The health promotion community needs to play a proactive role in shifting deficit-focused narratives about BYMOC, to one which has an explicit strengths-based orientation. There is substantial evidence to suggest that popular media depictions of BYMOC often have negative connotations or are aligned with hyper masculine traits, which can perpetuate racist and prejudicial attitudes, and a perceived ‘disposability of Black men’s lives’ (Goodwill *et al.*, 2019). Some organizations and programmes such as Forward Promise, the Campaign for Black Male Achievement and Making Connections have emphasized the importance of narratives focused on promise, success, achievement and life chances. They have shown that these concepts can easily be embedded into contemporary health promotion work with BYMOC. Ideally, this approach should be a central feature of all work with BYMOC across the USA. Examples include, but are not limited to, publicly celebrating key life milestones such as school and university graduations, transitions into work, sporting achievements and contributions to community leadership and volunteering; using positive imagery of BYMOC in health-related social marketing endeavours; and recognising the positive roles BYMOC play in families and communities as fathers, partners, brothers, uncles, friends, colleagues, coaches, teachers and mentors.

### Acknowledging both inclusiveness and diversity

Within a US context, BYMOC is intended to be an inclusive term. It includes a broad range of minority populations including African American, Native American, Latinx, Hispanic and Asian boys and young men. While promoting inclusiveness is important, and promotes a collective sense of belonging, it is vital to acknowledge the diversity and heterogeneity within and between sub-populations of BYMOC. While many of the health and social inequities BYMOC experience are similar and frequently underpinned by racial inequities, the historical, socio-political and cultural basis of these inequities can be different. Understanding these differences is critical for a few different reasons. First, appropriately tailored health promotion and public health responses need to be fit for purpose. Strategies that acknowledge local environmental, geographical and historical influences, and which unpack context-specific patterns of inequities among BYMOC are more likely to succeed. Second, conceptualizations of health and well-being are different among sub-populations of BYMOC. For example, Native American boys and young men may draw on cultural and spiritual notions of social and emotional well-being, or concepts of family, that have more holistic understandings of health, that may differ markedly from other populations that are also considered to be BYMOC. This has obvious implications for the development and delivery of health promotion interventions for these sub-populations, particularly interventions relating to mental health. Third, the collective framing of BYMOC influences policy development and implementation, and subsequent funding parameters. This can be problematic for addressing more specific racial, ethnic and cultural needs of sub-populations that fall under this broader umbrella category. Specifically, funding criteria directed towards programmes and services for BYMOC may not be sufficiently flexible to cater to these diverse needs. This inevitably means health promotion interventions targeting BYMOC are too often tailored to meet the needs of funders, rather than those of the community they are supposed to serve. In this sense, a sharper focus on the structural barriers created through grant funding criteria and parallel commissioning processes is vital, if the health promotion profession truly wants to reduce inequities among BYMOC.

### Addressing racism and intergenerational trauma

Racism and intergenerational trauma are critical social determinants of health (Bailey *et al.*, 2017; Ford *et al.*, 2019). Addressing their impacts should be foci of all health promotion interventions, programmes, and services targeting BYMOC. The structural drivers that underpin racism at a societal level, such as white privilege and white supremacy, must be acknowledged, challenged and transformed. The health promotion community has played an important role in raising awareness of these structural drivers, but more needs to be done. A more concerted focus on actions and practical strategies to remove structural and systemic barriers aimed at reducing inequities experienced by all people of colour would dramatically change the way work with BYMOC is done. This means calling out racial prejudice and highlighting programmes and services that promote healing, because these are legitimate public health strategies for acknowledging past and ongoing trauma (The National Compadres Network, 2012). Within the context of BYMOC this means acknowledging how the social construction of gender is intricately tied to concepts of race and trauma. While there is substantial academic scholarship about the need to challenge harmful masculine norms (Heilman *et al.*, 2017; Ragonese *et al.*, 2019), there is less—but growing—guidance about what more positive messaging can look like. We must also be aware of the unique nature of Black and Indigenous masculinities when developing health promotion actions for BYMOC, and how these connect with their conceptualizations of manhood (Metzl, 2013; Goodwill *et al.*, 2019). There are some promising examples of interventions and curricula that do this well (Goodwill *et al.*, 2018; Watkins *et al.*, 2017; Watkins *et al.*, 2020; The National Compadres Network, 2012). For example, the Young Black Men, Masculinities and Mental Health (YBMen) project is an education and social support programme that facilitates online discussions about mental health and manhood using prompts from popular culture and current news headlines (Goodwill *et al.*, 2018; Watkins *et al.*, 2017); and the National Compadres Network (2012) has adopted *La Cultura Cura* as a transformative Indigenous health and healing philosophy in its programmatic work with BYMOC.

### Committing to a national boys and men’s health policy

At present, the USA does not have a national men’s health policy that provides a clear roadmap for action. The American Public Health Association, the Men’s Health Network and the American Psychological Association have all developed documents that could guide such work [Giorgianni *et al.*, 2013; Nolan and Fadich, 2013; American Psychological Association (APA), 2018]. In countries where national men’s health policies have been developed and implemented, there has been a focus on equity and diversity to address the needs of the most marginalized and vulnerable groups of men (Richardson and Smith, 2011; Richardson *et al.*, 2019). For example, the Australian National Men’s Health Strategy includes an explicit principle to ‘ensure that equity drives investment and action’ [(Department of Health, 2019), p. 26], with an accompanying national framework and set or principles for improving the health and well-being of Aboriginal and Torres Strait Islander males (Department of Health and Ageing, 2010). A similar model could be adopted in the USA with respect to BYMOC.

## CONCLUSION

We have identified eight overarching challenges and opportunities faced by the public health community when attempting to reduce health and social inequities experienced by BYMOC. This is not meant to be an exhaustive list. Rather, we hope our commentary will stimulate further conversations about meeting the health, social, and cultural needs of BYMOC. We trust it will inform future health promotion planning, delivery and evaluation; guide public health policy development and implementation; enhance commissioning processes; generate new and innovative research ideas; and promote more purposeful partnership development. We encourage health promotion researchers, practitioners and policy-makers to adopt the solutions we offer to help reduce the structural and systemic inequities experienced by BYMOC to ensure they can achieve optimal health and wellbeing.
